# Nickelocene-Filled Purely Metallic Single-Walled Carbon Nanotubes: Sorting and Tuning the Electronic Properties

**DOI:** 10.3390/nano11102500

**Published:** 2021-09-26

**Authors:** Marianna V. Kharlamova

**Affiliations:** 1 Institute of Materials Chemistry, Vienna University of Technology, Getreidemarkt 9/BC/2, 1060 Vienna, Austria; mv.kharlamova@gmail.com; 2 Moscow Institute of Physics and Technology, Institutskii Pereulok 9, 141700 Dolgoprudny, Russia

**Keywords:** single-walled carbon nanotube, nickelocene, electronic properties, X-ray photoelectron spectroscopy, Raman spectroscopy

## Abstract

We conducted the filling of single-walled carbon nanotubes (SWCNTs) with nickelocene molecules and separation of the filled SWCNTs by conductivity type by density-gradient ultracentrifugation. We tailored the electronic properties of nickelocene-filled purely metallic SWCNTs by thermal treatment in high vacuum. Our results demonstrated that annealing at low temperatures (360–600 °C) leads to n-doping of SWCNTs, whereas annealing at high temperatures (680–1200 °C) results in p-doping of SWCNTs. We found a correlation between the chemical state of the incorporated substances at different annealing temperatures and its influence on the electronic properties of SWCNTs.

## 1. Introduction

Carbon nanotubes, one-dimensional allotropic modifications of carbon with sp^2^-hybridization of atoms, can be envisaged as graphene sheets rolled into cylinders. Depending on the number of graphene layers, the nanotubes are classified as single- (SWCNT), double- (DWCNT) and multi-walled. SWCNTs are attracting the attention of researchers thanks to their unique physical and chemical properties, which can be applied in different fields. SWCNTs are promising materials for the next generation of nanoelectronic devices [1]. The properties of SWCNTs are defined by their atomic structure. Contemporary synthesis methods allow preparing the mixture of nanotubes with different structures and properties, limiting their application [2].

To obtain SWCNTs with defined electronic properties, two approaches were developed. The first approach is the separation of synthesized SWCNTs by conductivity type and chiral vector [3,4]. The second approach is the modification of the properties of SWCNTs via covalent and noncovalent functionalization of the outer surface of SWCNTs, substitution of atoms in the SWCNT walls by other atoms, intercalation of the bundles of SWCNTs and the filling of the internal channels of SWCNTs [5]. The filling of SWCNTs is a promising method of controllable modification of their electronic properties because substances with different physical and chemical properties can be incorporated inside SWCNTs [6]. In the literature, there are examples of the filling of SWCNTs with electron donors—metals, which lead to raising the Fermi level of SWCNTs [7,8]—and electron acceptors—metal halogenides, which result in lowering the Fermi level of SWCNTs [9,10,11]. The filling of SWCNTs separated by conductivity type to metallic and semiconducting fractions offers an opportunity to precisely tailor their electronic structure. In the literature, there are only a few examples of filling of metallic and semiconducting SWCNTs by chlorides of silver [12,13] and copper [14].

The filled metallic and semiconducting SWCNTs can be obtained not only by the filling of SWCNTs separated by conductivity type but also by the separation of the filled SWCNTs. Recently, the authors of [15] performed the separation of nickelocene-filled SWCNTs to metallic and semiconducting fractions by density gradient ultracentrifugation. This approach has advantages, because it allows not only separating the filled SWCNTs but also cleaning them from non-encapsulated substances.

Metallocene-filled SWCNTs are of special interest because it has been shown that the thermal treatment in a vacuum of filled SWCNTs leads to the formation of inner nanotubes [16]. Annealing of filled SWCNTs results in the decomposition of metallocene with the formation of metal carbide or metal, which act as catalysts for the growth of inner nanotubes [16]. The chemical transformation of metallocene and the growth of inner tubes can lead to the modification of the electronic properties of SWCNTs. This gives an opportunity to tailor the electronic structure of metallocene-filled SWCNTs by annealing at different temperatures. At the same time, there are no reports in the literature on studies of the thermal treatment-induced modification of the electronic properties of metallocene-filled metallic SWCNTs separated by the conductivity-type.

In this study, we close this gap by investigating the electronic properties of nickelocene-filled high-purity metallic SWCNTs annealed at temperatures between 360 and 1200 °C by X-ray photoelectron spectroscopy (XPS). The chemical state of the filler at different annealing temperatures is studied by XPS. The growth process of inner tubes is investigated by Raman spectroscopy. On the basis of the obtained data, we found a correlation between the chemical state of the incorporated substances and its influence on the electronic properties of SWCNTs.

## 2. Materials and Methods

SWCNTs with mixed-conductivity type and a mean diameter of 1.67 nm were used for filling with nickelocene. The SWCNTs were synthesized by the chemical vapor deposition method [17]. Prior to filling, the ends of the SWCNTs were opened by annealing in air at 500 °C for 1 h. Then, the SWCNTs and an excess of nickelocene powder (NiCp_2_, chemical formula (C_5_H_5_)_2_Ni, 99%, Strem Chemicals Inc., Bischheim, France) were put into Pyrex-glass ampoules. The ampoules were evacuated at a pressure of 10^−^^6^ mbar for 20 min and sealed. Half of the ampoule was heated up to a temperature of 50 °C; this led to the evaporation of nickelocene and its condensation in the colder half of the ampoule. Depending on the amount of nickelocene, this process took between 12 and 24 h. After that, the position of the ampoule was flipped so that the nickelocene containing half of the ampoule was heated. This procedure was repeated 5–10 times over 5 days. Then, the filled SWCNTs were separated into metallic and semiconducting fractions by density-gradient ultracentrifugation. The details of the separation procedure are described in Reference [15]. The filled metallic SWCNTs were annealed in a high vacuum (10^−6^ mbar) at temperatures between 360 and 1200 °C for 2 h.

The investigation of the samples by XPS was performed with a VG Scienta XPS spectrometer using monochromatic Al K_ radiation with an energy of 1486.6 eV and semispherical photoelectron analyzer SCIENTA RS4000. The samples, in the form of buckypapers with a size of 5 × 5 mm, were mounted on Mo holders. The measurements were conducted at room temperature. The calibration of the energy scale was performed using the position of the Au 4f_7__/__2_ peak at 83.96 eV. The investigation of the samples by Raman spectroscopy was conducted with a Horiba Jobin Yvon LabRAM HR800 spectrometer using a laser wavelength of 514 nm (ArKr, Coherent Innova 70c, Dieburg, Germany). The measurements were performed directly on the SWCNT buckypapers at room temperature. The fitting of the obtained spectra with Voigtian functions was performed with PeakFit 4.12. The accuracy in the peak positions is ±2 cm^−^^1^.

## 3. Results

**Figure 1:**
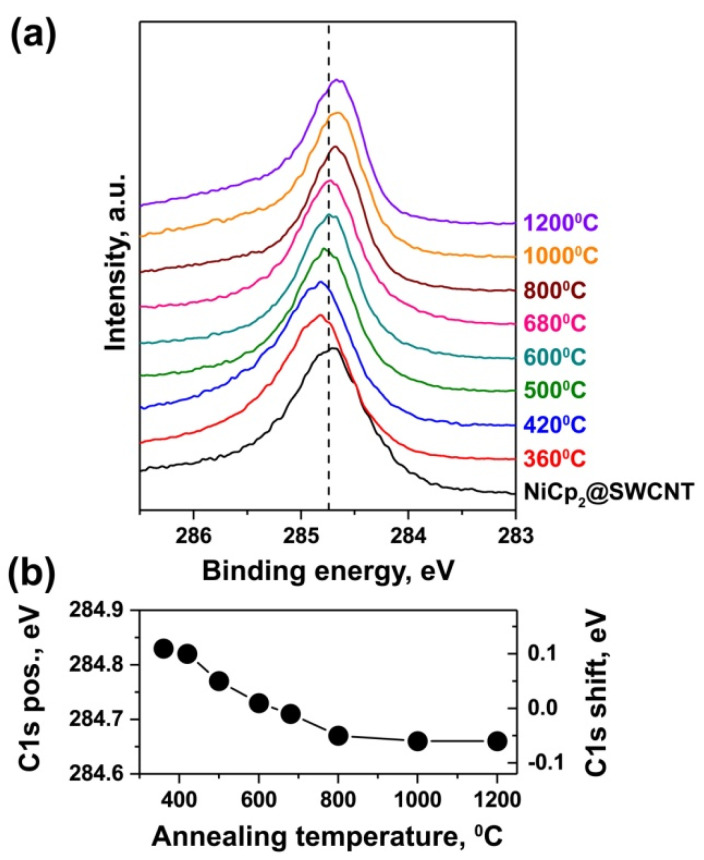
(**a**) The C1s XPS spectra of nickelocene-filled SWCNTsand samples annealed at temperatures between 360 and 1200 °C for 2 h. (**b**) The dependence of the position of the C1s peak and its shift relative to the position for unannealed sampleson annealing temperatures.

Figure 1a shows the C 1s XPS spectra of nickelocene-filled SWCNTs and samples annealed at temperatures between 360 and 1200 °C for 2 h. The spectra of all samples include a single peak, which is positioned at a binding energy of 284.73 eV for nickelocene-filled SWCNTs. The annealing of the filled SWCNTs leads to the shift of the peak. Figure 1b presents the dependence of the peak position and its shift relative to the position of the unannealed sample on the annealing temperature. The annealing at a temperature of 360 °C leads to the shift of the peak by 0.11 eV towards higher binding energies. As annealing temperatures increase, the peak shifts gradually towards lower binding energies and almost reaches the position of the unannealed sample at 600 °C. The annealing at a temperature of 800 °C leads to the further shift of the peak by 0.04 eV. As annealing temperatures increase, the peak position changes insignificantly, and the maximal shift of the peak of 0.06 eV is observed at a temperature of 1200 °C. The observed shift of the peak towards higher binding energies during annealing at low temperatures (360–600 °C) and towards lower binding energies during annealing at high temperatures (680–1200 °C) is caused by the modification of the electronic properties of SWCNTs.

According to the literature data, the shift of the C1s XPS peak towards higher binding energies was observed for SWCNTs intercalated by alkali metals [18] and filled with ferrocene [19] and europium [20]. The appearance of additional components at higher binding energies as compared to the peak of the pristine SWCNTs was observed in the C 1s XPS spectra of SWCNTs filled with silver and copper [7,8]. This was explained by the charge transfer from encapsulated substances to the SWCNTs, i.e., n-doping of SWCNTs. The shift of the C1s XPS peak toward lower binding energies was observed for SWCNTs filled with metallofullerene Gd@C82 [21], cerocene molecules [22] and erbium chloride [23]. The appearance of additional components at lower binding energies as compared to the peak of the pristine SWCNTs was observed in the C1s XPS spectra of SWCNTs filled with metal halogenides [9,10,11]. This was explained by the charge transfer from the SWCNTs to encapsulated substances, i.e., p-doping of SWCNTs. Taking into consideration this interpretation of the shifts of the peaks in the C1s XPS spectra, we can conclude that the annealing of nickelocene-filled SWCNTs at low temperatures (360–600 °C) leads to n-doping of SWCNTs, whereas the annealing at high temperatures (680–1200 °C) results in p-doping of SWCNTs.

**Figure 2:**
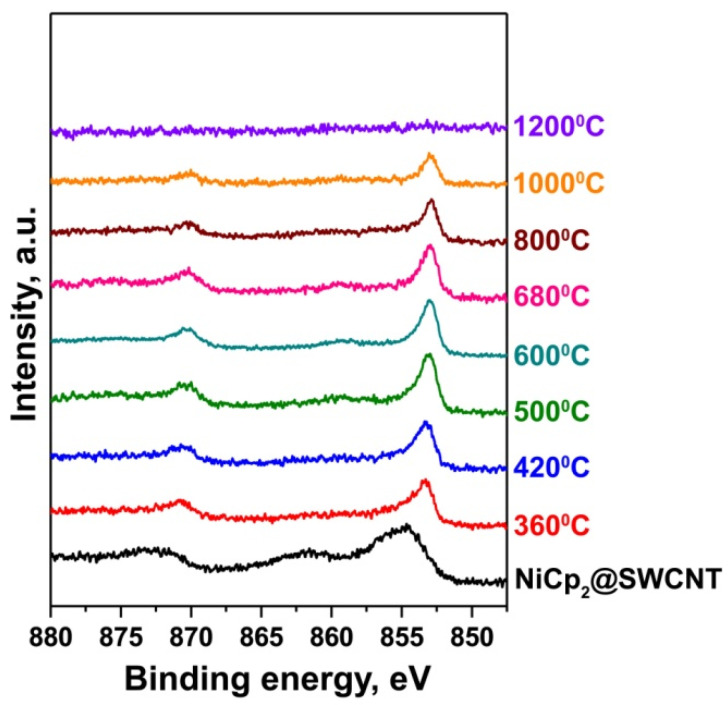
The Ni 2p XPS spectra of nickelocene-filled SWCNTs and samples annealed at temperatures between 360 and 1200 °C for 2 h.

In order to explain the modification of the electronic properties of SWCNTs observed with the thermal treatment of nickelocene-filled SWCNTs, we investigated the chemical state of the filler at different annealing temperatures. Figure 2 shows the Ni 2p XPS spectra of SWCNTs filled with nickelocene and samples annealed at temperatures of 360–1200 °C for 2 h. The spectrum of nickelocene-filled SWCNTs includes two broad peaks positioned at binding energies of 854.7 and 872.2 eV; they belong to Ni 2p_3__/__2_ and Ni 2p_1__/__2_ core levels. The peak positions are close to the ones of pure nickelocene [24]. These peaks have an asymmetric shape because of the presence of shoulders at binding energies of 856.1 and 873.7 eV. They belong to oxidized nickel, which was formed during the treatment of nickelocene-filled SWCNTs with chemicals in the process of separation.

During annealing at a temperature of 360 °C, the Ni 2p_3__/__2_ and Ni 2p_1__/__2_ peaks shift by 1.4 eV toward lower binding energies. This is explained by the change in the chemical state of nickel as a result of the decomposition of nickelocene with the formation of nickel carbide. It is not clear whether the carbon atoms needed to form the carbide arose from the SWCNTs (perhaps from the inner tubes) or from the ligands of the nickelocene molecules.With increasing annealing temperatures, the Ni 2p_3__/__2_ and Ni 2p_1__/__2_ peaks are narrowed and further shift towards lower binding energies, reaching the position of metallic nickel (for which the Ni 2p_3__/__2_ peak is positioned at a binding energy of ~853 eV [25]) at 600 °C. This data proves that metastable nickel carbides transform to stable metallic nickel. This conclusion is in agreement with the reported results that showed that nickel carbides (in particular, Ni3C) are metastable and decompose at temperatures higher than 400–500 °C [26,27]. With annealing temperatures increasing higher than 600 °C, the intensity of the Ni 2p3/2 and Ni 2p_1__/__2_ peaks gradually decreases; this corresponds to releasing nickel from the nanotube channels until its complete removal during annealing at the maximal temperature of 1200 °C. A similar trend was observed during the annealing of ferrocene-filled SWCNTs [16].

Regarding the Ni concentration, there is a high nickel content in the nickelocene-filled SWCNTs. The atomic ratio of nickel-to-carbon amounts to 0.0141. The annealing of the filled SWCNTs leads to the removal of nickel from the sample. At 500 °C, the atomic ratio of nickel-to-carbon amounts to 0.0100, which corresponds to a nickel content of 71%. At 800 °C, the atomic ratio of nickel-to-carbon amounts to 0.0047, which corresponds to a nickel content of 33%. Finally, at 1200 °C, the atomic ratio of nickel-to-carbon amounts to 0.0004, which corresponds to a nickel content of 3%.

**Figure 3:**
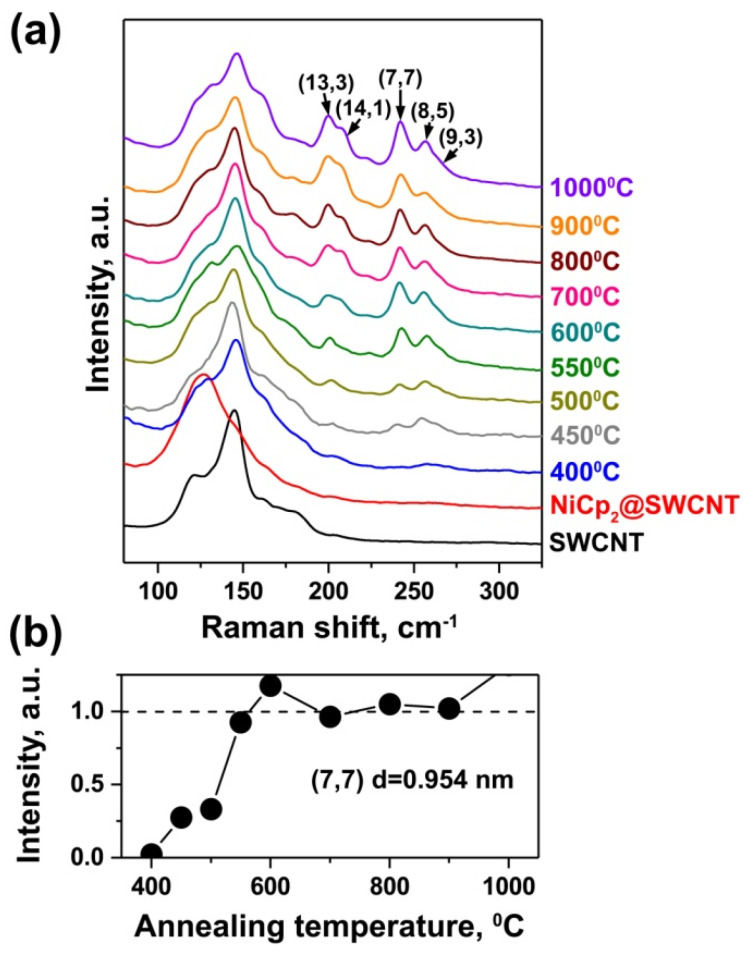
(**a**) The RBM-band of Raman spectra of SWCNTs, nickelocene-filled SWCNTs and samples annealed at temperatures between 400 and 1000 °C for 2 h. The chiral vectors of inner nanotubes are denoted. (**b**) The dependence of normalized intensity of the peak of inner tube with chirality of (7,7) on annealing temperature.

Not only the thermal treatment-induced chemical transformation of nickelocene but also the growth process of inner nanotubes influences the electronic properties of the host SWCNTs. To investigate the growth process of inner tubes, the samples were studied by Raman spectroscopy. Figure 3a shows the radial breathing mode (RBM) bands of Raman spectra of the pristine SWCNTs, nickelocene-filled SWCNTs and samples annealed at temperatures of 400–1000 °C for 2 h. The RBM-band of the pristine SWCNTs includes an intense peak at 145 cm^−1^, which corresponds to the nanotubes with a diameter of 1.7 nm [28]. The RBM-band of nickelocene-filled SWCNTs includes the peak shifted by 19 cm^−^^1^ towards lower frequencies, which is common for molecule-filled SWCNTs [16]. The spectra of the annealed samples show the appearance of additional peaks of inner tubes. These peaks are positioned at frequencies of 198, 207, 242, 255 and 263 cm^−^^1^ and, according to the Kataura plot [29], correspond to the nanotubes with chiralities of (13,3), (14,1), (7,7), (8,5) and (9,3) and diameters of 1.16, 1.14, 0.95, 0.90 and 0.85 nm, respectively. Taking into consideration the mean diameter of the pristine SWCNTs (1.67 nm) and doubled van der Waals distance between graphene layers of DWCNTs (0.67 nm), we conclude that the mean diameter of inner nanotubes amounts to 1.0 nm. Figure 3b shows the dependence of normalized area intensity of the peak of the (7,7) tube with the mean diameter on the annealing temperature. It is visible that the intensity of the peak of this tube increases at temperatures between 450 and 550 °C and stays unchanged at higher annealing temperatures. Consequently, the sample consists of SWCNTs and DWCNTs at temperatures of 450–550 °C, and it contains filled or empty DWCNTs at temperatures higher than 550 °C.

**Figure 4:**
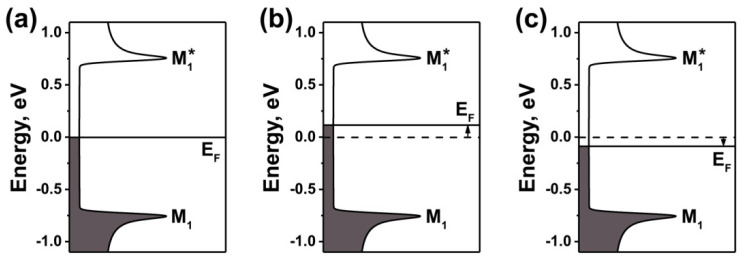
The schematics of the band structures of metallic SWCNTs (**a**) and nickelocene-filled SWCNTs annealed at temperatures of 360–600 °C (**b**) and 680–1200 °C (**c**) for 2 h. E_F_ is the Fermi level, and M1 and M1* are the first vHs in the valence and conduction band, respectively. The Fermi level shift is indicated by arrows.

Thus, there are three overlapping temperature-dependent processes that influence the electronic properties of SWCNTs: (i) chemical transformation of nickelocene, (ii) inner tube growth and (iii) removal of nickel from the nanotubes. These materials might be expected to behave in a manner that is consistent with prior theoretical and experimental studies of analogous materials [19]. During annealing at low temperatures, nickelocene transformed to nickel carbides and metallic nickel, which led to n-doping of SWCNTs. During annealing at higher temperatures, inner tube growth and removal of filler from the nanotubes occurred, which led to the formation of empty DWCNTs. In the DWCNTs, p-doping of the outer tubes by inner tubes was present, which is in agreement with the data of theoretical modeling [30]. Figure 4 shows the schematics of the band structures of the pristine metallic SWCNTs and the Fermi level shifts in nickelocene-filled SWCNTs annealed at temperatures between 360 and 1200 °C.

## 4. Conclusions

In conclusion, we tailored the electronic properties of nickelocene-filled purely metallic SWCNTs by thermal treatment in a high vacuum. It was shown that annealing at low temperatures (360–600 °C) led to n-doping of SWCNTs, whereas annealing at high temperatures (680–1200 °C) resulted in p-doping of SWCNTs.

## Data Availability

Not applicable.
